# Alleviation of adverse effects of drought stress on wheat seed germination using atmospheric dielectric barrier discharge plasma treatment

**DOI:** 10.1038/s41598-017-16944-8

**Published:** 2017-11-30

**Authors:** Qiao Guo, Ying Wang, Haoran Zhang, Guangzhou Qu, Tiecheng Wang, Qiuhong Sun, Dongli Liang

**Affiliations:** 1 State Key Laboratory of Soil Erosion and Dryland Farming on the Loess Plateau, Institute of Soil and Water Conservation, Northwest A&F University, Shaanxi Province, 712100 P.R. China; 2 College of Natural Resources and Environment, Northwest A&F University, Yangling, Shaanxi Province 712100 P.R. China; 3 Key Laboratory of Plant Nutrition and the Agri-environment in Northwest China, Ministry of Agriculture, Yangling, Shaanxi 712100 P.R. China

## Abstract

Atmospheric dielectric barrier discharge (DBD) was attempted to improve the resistance of wheat seed to drought stress. Effects of DBD plasma on wheat seed germination, seedling growth, osmotic-adjustment products, lipid peroxidation, reactive oxygen species (ROS), antioxidant enzyme activity, abscisic acid, and drought resistant related genes expression under drought stress were investigated. The changes of the wheat seed coat before and after the DBD plasma treatment were explored. Experimental results showed that the DBD plasma treatment could alleviate the adverse effects of drought stress on wheat seed germination and seedling growth; the germination potential and germination rate increased by 27.2% and 27.6%, and the root length and shoot length of the wheat seedlings also increased. Proline and soluble sugar levels under drought stress were improved after the DBD plasma treatment, whereas the malondialdehyde content decreased. ROS contents under drought stress were reduced after the DBD plasma treatment, whereas the activities of superoxide dismutase, catalase, and peroxidase were promoted. DBD plasma treatment promoted abscisic acid generation in wheat seedlings, and it also regulated functional gene *LEA1* and stimulated regulation genes *SnRK2* and *P5CS* to resist drought stress. Etching effect and surface modification occurred on the seed coat after the DBD plasma treatment.

## Introduction

With the continuous deterioration of global warming, water resource deficits and the uneven distribution of water in the world has already resulted in severe water shortages in many countries. Drought, as one of the most severe environmental problems caused by water shortages, has become a major limiting factor on crop production in the majority of the agricultural fields of the world^1^. Drought stress could inhibit the growth of crops, via production of a variety of changes in physiological, biochemical, morphological, and molecular behaviors in plants^2^. Wheat is one of the major food staples worldwide, and its yield is unavoidably affected by water resource shortages in the environment^3^. Presently, wheat is the largest food staple in Northwest China, this region has a broad semiarid area, and drought is the major factor for losses of wheat yield^4^. Therefore, it is quite important to alleviate the adverse effects of drought stress on wheat growth.

Improving the drought resistance of seeds is an effective pathway to alleviate the adverse effects of drought stress. Several methods have been employed to improve the drought resistance of the crop seed such as hormonal regulation^5^, drought hardening^6^, seed soaking^7^, silicon application^8^, magnetic field treatment^9^, and electric field treatment^10^. Among these methods, more concern has been given to physical methods, such as magnetic field and electric field treatments, due to their weak damage to the seeds and the near absence of chemical residue. As one of the physical methods, cold plasma is considered an economical and safe approach for seed treatment, and it is composed of ionized gases, radicals, excited atoms, molecules, electrons, a strong electric field, and ultraviolet irradiation, which could result in stimulating effects on plants^11–14^. Recently, cold plasma has been proven to improve seed germination and growth, for example in *tomato*, *wheat* and *oat* ^11–13^; and it could also improve the activities of superoxide dismutase (SOD), catalase (CAT), and peroxidase (POD) and decrease the lipid peroxidation (MDA) content of *Andrographis paniculata* seedlings^15^, which indicated an increase in the tolerance to environmental stress. More importantly, Li *et al*. reported that a cold plasma treatment enhanced oilseed rapeseed germination under drought stress; however, a high plasma frequency (13.56 MHz) and low gas pressure (150 Pa) were needed in such a system^16^.

Dielectric barrier discharge (DBD) plasma was one type of cold plasma that could be easily triggered at atmospheric pressure and room temperature^17^. DBD plasma could generate ultraviolet radiation, a strong electric field, electrons, and various active species, and no rare gas source or vacuum equipment was needed. It was reported that DBD plasma treatment improved seed germination using a high frequency and high voltage alternating current power supply at a frequency of approximately 8.0 ~ 15.4 kHz^18,19^. Compared with the high frequency and high voltage alternating current power supply with a frequency of approximately 8.0 ~ 15.4 kHz, it was much easier to manufacture the high voltage alternating current power supply with a fixed-frequency of 50 Hz, because the alternating current power supply with a fixed-frequency of 50 Hz has been widely used in the production and daily life in China; therefore, in our previous research, DBD plasma with a fix-frequency of 50 Hz was tried to treat wheat seed, and the wheat seed germination and seedling growth were enhanced and the permeability of seed coat and soluble protein content were also improved after the DBD plasma treatment^20^. However, it was unclear whether a DBD plasma treatment with a fix-frequency of 50 Hz could improve the drought resistance of wheat seed.

Therefore, the aim of this study was to examine whether the DBD plasma treatment with a fix-frequency of 50 Hz could alleviate the adverse effects of drought stress on wheat seed germination. Here, the effects of DBD plasma treatment on the wheat seed germination and seedling growth under drought stress were evaluated; the underlying regulatory mechanisms for drought resistance enhancement were investigated via the changes of the osmotic adjustment ability, membrane lipid peroxidation, reactive oxygen species (ROS), abscisic acid (ABA), and antioxidant enzyme activities of the wheat seedlings. In addition, the possible actions of the DBD plasma on the wheat seed coat were also explored via Scanning electron microscope (SEM), Fourier transform infrared spectroscopy (FTIR), and Energy dispersive X-ray spectrum (EDX) analysis. This was expected to provide a theoretical and practical foundation for the application of DBD plasma in the improvement of seed drought resistance.

## Results

### Wheat seed germination and seedling growth under drought stress before and after DBD plasma treatment

The results of wheat seed germination under drought stress after the DBD plasma treatment are shown in Table 1. The DBD plasma treatment promoted the wheat seed germination under drought stress; the mean germination potential and germination rate were both significantly enhanced after the DBD plasma treatment compared with those without the DBD plasma treatment. For instance, there was approximately 17.3% increase in the germination rate in the “plasma + drought stress” group, compared to that in the “drought stress” group. The germination index of drought resistance was also enhanced by 15.1% after the DBD plasma treatment.

**Table 1:** Wheat seed germination and seedling growth under drought stress before and after DBD plasma treatment. (The discharge voltage is 13.0 kV, and the DBD plasma treatment time is 4 min. GP is sampled on the 1^st^ day of planting. GR_4_, root length, and shoot length are sampled on the 4^th^ day of planting. GI and GIDR are sampled on the 8^th^ day of planting. Data are presented as the mean ± SD (n = 3). Different lower case letters (a-d) indicate statistically significant differences at the *P* < 0.05 level).

Treatment groups	GP (%)	GR_4_ (%)	GI	GIDR	Root length (mm)	Shoot length (mm)
watered	62.5 ± 6.1b	88.0 ± 3.5b	2.120 ± 0.041b	—	1573.7 ± 79.8b	468.8 ± 5.6b
plasma + watered	77.5 ± 4.3a	95.3 ± 3.2a	2.346 ± 0.058a	—	1759.3 ± 46.4a	490.9 ± 5.1a
drought stress	39.3 ± 3.1d	62.7 ± 3.1d	1.502 ± 0.045d	0.708	727.0 ± 24.2d	153.1 ± 12.3d
plasma + drought stress	50.0 ± 4.0c	80.0 ± 2.0c	1.912 ± 0.063c	0.815	872.3 ± 62.3c	201.9 ± 12.2c

The results of wheat seedling growth under drought stress after the DBD plasma treatment are also shown in Table 1. The DBD plasma treatment promoted wheat seedling growth under the drought stress; the mean root length and shoot length were both significantly improved after the DBD plasma treatment, compared with those without the DBD plasma treatment. For instance, the mean root length and shoot length increased by 20.0% and 31.9%, respectively, in the “plasma + drought stress” group compared to those in the “drought stress” group.

The wheat seed germination photos under drought stress after the DBD plasma treatment are exhibited in Fig. S1. As can be seen, the best growth morphology was observed in the “plasma + watered” group, and the worst occurred in the “drought stress” group. In addition, significantly positive correlations were observed among germination potential, germination rate, root length, and shoot length in seedlings under experimental treatments, whereas these parameters were negatively correlated with ROS contents (Table 2).

**Table 2:** Pearson’s correlation coefficients among germination potential (GP), germination rate (GR), root length (RL), shoot length (SL), soluble sugar, proline, MDA, H_2_O_2_, O_2_^−^, SOD, POD, CAT, and ABA under drought stress and DBD plasma treatment (*^,^ **significantly different at the 5% and 1% probability levels, respectively).

	GP	GR	RL	SL	Soluble sugar	Proline	MDA	H_2_O_2_	O_2_^−^	SOD	POD	CAT	ABA
GP	1												
GR	0.957*	1											
RL	0.960*	0.912*	1										
SL	0.932*	0.928*	0.995**	1									
Soluble sugar	−0.592	−0.553	−0.795	−0.842	1								
Proline	−0.477	−0.494	−0.698	−0.762	0.975*	1							
MDA	−0.942	−0.916	−0.996**	−0.999**	0.822	0.743	1						
H_2_O_2_	−0.986*	−0.982*	−0.972*	−0.959*	0.660	0.576	0.969*	1					
O_2_^−^	−0.959*	−0.956*	−0.988*	−0.987*	0.763	0.692	0.993**	0.989*	1				
SOD	−0.503	−0.495	−0.723	−0.782	0.990*	0.996**	0.762	0.591	0.705	1			
POD	−0.275	−0.231	−0.534	−0.600	0.936	0.947	0.570	0.355	0.489	0.957*	1		
CAT	−0.352	−0.364	−0.596	−0.668	0.953*	0.990*	0.645	0.455	0.584	0.985*	0.975*	1	
ABA	−0.472	−0.368	−0.693	−0.735	0.963*	0.911	0.706	0.513	0.623	0.942	0.957*	0.913	1

### Proline and soluble sugar levels under drought stress before and after DBD plasma treatment

**Figure 1:**
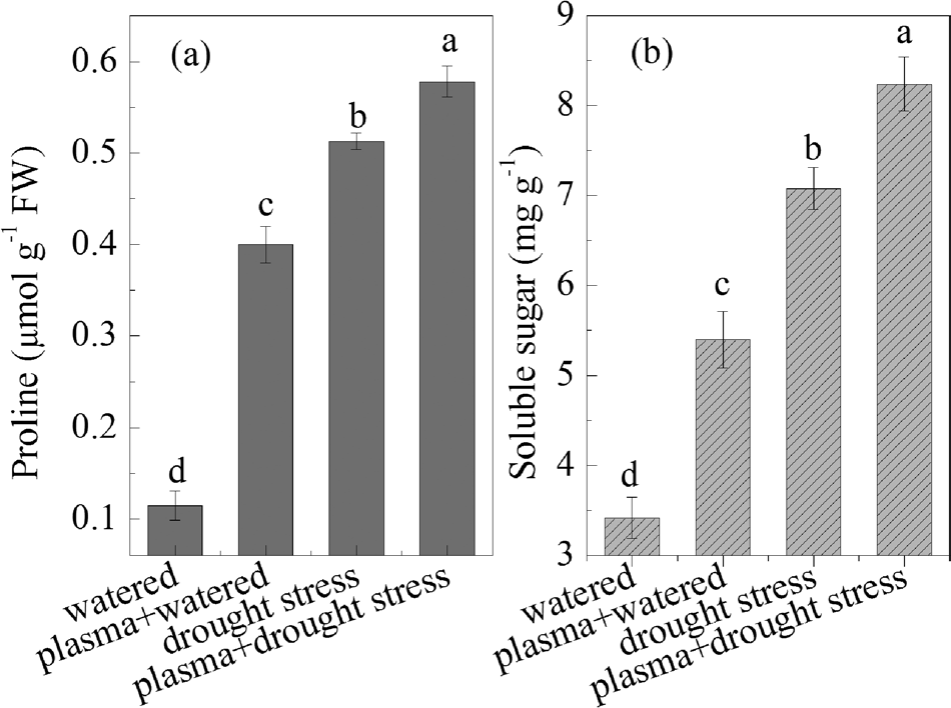
Effects of drought stress and DBD plasma treatment on proline and soluble sugar contents. (**a)** proline; (**b**) soluble sugar. The discharge voltage is 13.0 kV, and the DBD plasma treatment time is 4 min. Samples are collected on the 4^th^ day of planting. Data are presented as the mean ± SD (n = 3). Different lower case letters (**a**–**d**) indicate statistically significant differences at the *P* < 0.05 level).

The results of wheat seedling proline and soluble sugar levels under drought stress after the DBD plasma treatment are shown in Fig. 1. The DBD plasma treatment promoted the proline and soluble sugar accumulation in the wheat seedlings under drought stress; for instance, there was an approximately 12.7% increase in the proline content in the “plasma + drought stress” group, compared to that in the “drought stress” group, as shown in Fig. 1a. In addition, the proline content significantly increased in the “drought stress” group, compared to the “watered” group. A significantly positive correlation was observed between the proline and soluble sugar, SOD, and CAT activities under the experimental treatments (Table 2).

However, the soluble sugar content was significantly improved after the DBD plasma treatment, as shown in Fig. 1b.; for instance, the soluble sugar content increased by 16.4% in the “plasma + drought stress” group, compared with that in the “drought stress” group. A significantly positive correlation was found between soluble sugar and proline, SOD, CAT, and ABA under the experimental treatments (Table 2).

### Seedling MDA levels under drought stress before and after DBD plasma treatment

**Figure 2:**
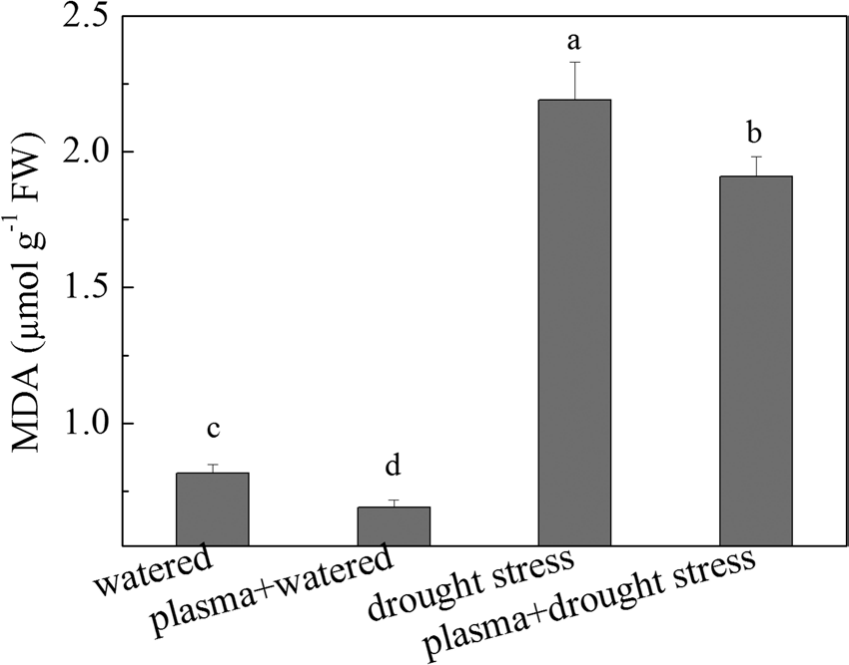
Changes in MDA content under drought stress before and after DBD plasma treatment. (The discharge voltage is 13.0 kV, and the DBD plasma treatment time is 4 min. Samples are collected on the 4^th^ day of planting. Data are presented as the mean ± SD (n = 3). Different lower case letters (**a**–**d**) indicate statistically significant differences at the *P* < 0.05 level).

The results of wheat seedling MDA levels under drought stress after the DBD plasma treatment are shown in Fig. 2. The DBD plasma treatment decreased the MDA accumulation in the wheat seedlings under drought stress. There was an approximately 12.8% and 15.3% decrease in the MDA content in the “plasma + drought stress” group and the “plasma + watered” group, respectively, compared to that in the “drought stress” group and the “watered” group. A significantly positive correlation was found between the MDA and ROS contents under the experimental treatments; whereas a negative correlation was observed between the MDA and germination and seedling growth (Table 2).

### Seedling ROS levels under drought stress before and after DBD plasma treatment

**Figure 3:**
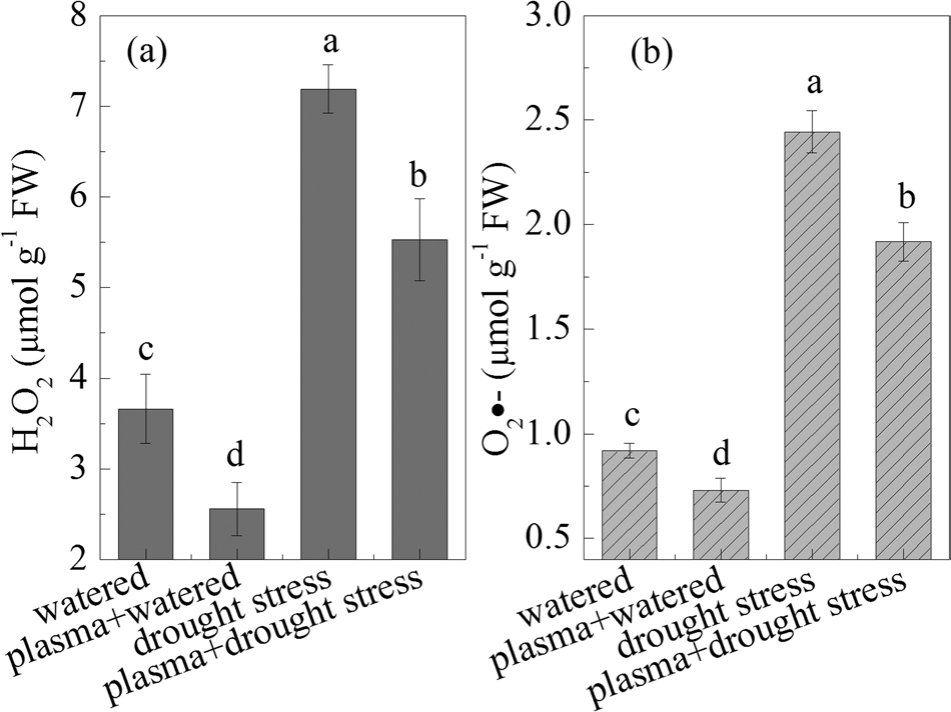
Changes of H_2_O_2_ and O_2_^−^ contents under drought stress before and after DBD plasma treatment. (**a**) H_2_O_2_; (**b**) O_2_^−^. The discharge voltage is 13.0 kV, and the DBD plasma treatment time is 4 min. Samples are collected on the 4^th^ day of planting. Data are presented as the mean ± SD (n = 3). Different lower case letters (**a**–**d**) indicate statistically significant differences at the *P* < 0.05 level).

The results of the wheat seedling H_2_O_2_ and O_2_^−^ levels under drought stress after the DBD plasma treatment are shown in Fig. 3. The DBD plasma treatment decreased the ROS levels in the wheat seedlings under drought stress. For instance, there was an approximately 23.1% decrease in the H_2_O_2_ content in the “plasma + drought stress” group, compared to that in the “drought stress” group, as shown in Fig. 3a.

However, the O_2_^−^ yield was significantly inhibited after the DBD plasma treatment, as shown in Fig. 3b. There was an approximately 21.5% and 20.6% decrease in the O_2_^−^ content in the “plasma + drought stress” group and the “plasma + watered” group, respectively, compared to that in the “drought stress” group and the “watered” group. In addition, a significantly positive correlation was found between the ROS contents and MDA under the experimental treatments; whereas a negative correlation was observed between the ROS contents and germination and seedling growth (Table 2).

### Seedling antioxidant enzyme levels under drought stress before and after DBD plasma treatment

**Figure 4:**
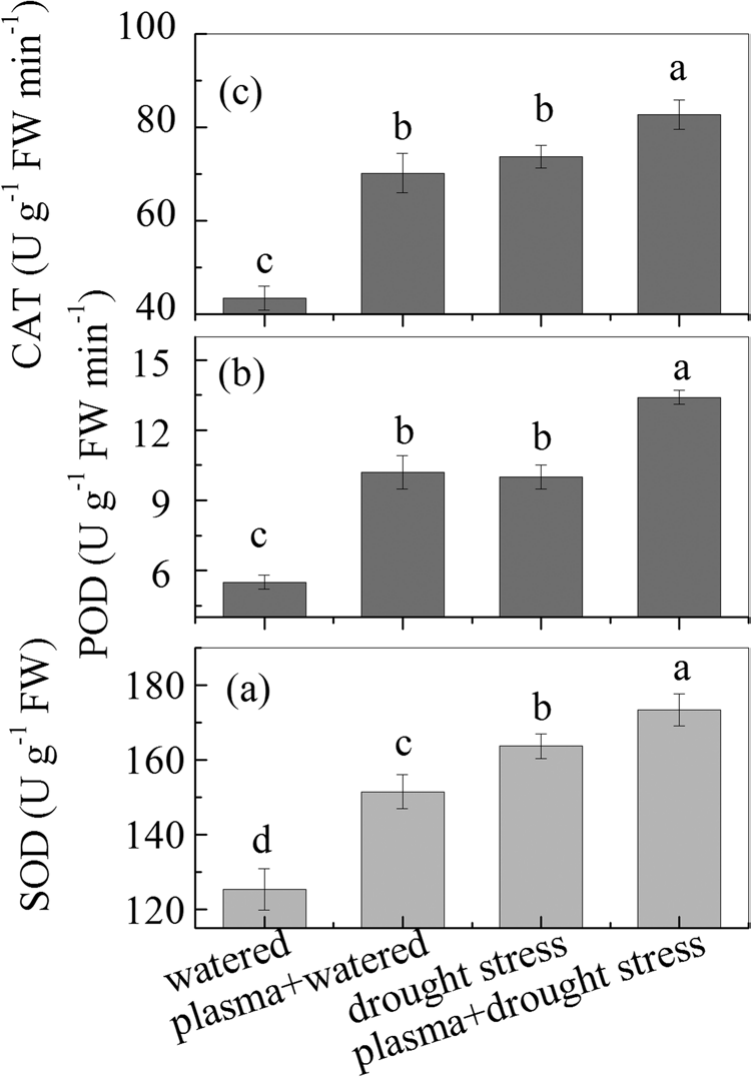
Evolution of enzyme activity under drought stress before and after DBD plasma treatment. (**a**) SOD; (**b**) POD; and (c) CAT. The discharge voltage is 13.0 kV, and the DBD plasma treatment time is 4 min. Samples are collected on the 4^th^ day of planting. Data are presented as the mean ± SD (n = 3). Different lower case letters (**a**–**d**) indicate statistically significant differences at the *P* < 0.05 level).

The results of wheat seedling antioxidant enzyme levels under drought stress after the DBD plasma treatment are shown in Fig. 4. As shown in Fig. 4a, the DBD plasma treatment promoted SOD activity in the wheat seedlings under drought stress. In addition, a significantly positive correlation was found between SOD and POD, CAT, soluble sugar, and proline under the experimental treatments (Table 2).

As shown in Fig. 4b, the POD activity was significantly improved after the DBD plasma treatment; for instance, there was an approximately 34% increase in the POD activity in the “plasma + drought stress” group, compared with that in the “drought stress” group. The mean POD activity also significantly increased in the “drought stress” group, compared to the “watered” group; and there was an approximately 81.8% increase in the POD activity in the “drought stress” group, compared to the “watered” group. In addition, a significantly positive correlation was found between POD and SOD, CAT, and ABA under the experimental treatments (Table 2). The CAT activity was also significantly improved after the DBD plasma treatment, as shown in Fig. 4c, In addition, a significantly positive correlation was found between CAT and SOD, POD, soluble sugar, and proline under the experimental treatments (Table 2).

### ABA levels under drought stress before and after DBD plasma treatment

**Figure 5:**
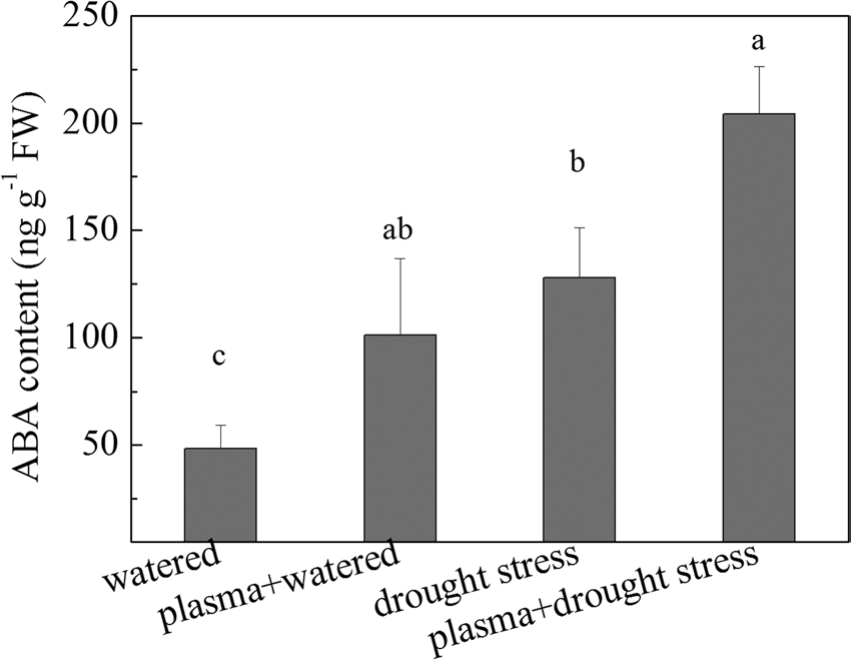
Evolution of ABA levels under drought stress before and after DBD plasma treatment. (The discharge voltage is 13.0 kV, and the DBD plasma treatment time is 4 min. Samples are collected on the 4^th^ day of planting. Data are presented as the mean ± SD (n = 3). Different lower case letters (**a**–**d**) indicate statistically significant differences at the *P* < 0.05 level).

The results of the ABA level under drought stress after the DBD plasma treatment are shown in Fig. 5. The DBD plasma treatment increased the accumulation of ABA and there was an approximately 37.9% increase in the ABA content in the “plasma + drought stress” group, compared to that in the “drought stress” group. However, the ABA content significantly increased in the “drought stress” group, compared to the “watered” group. A significantly positive correlation was found between ABA and POD, and soluble sugar under the experimental treatments (Table 2).

### Drought resistant related genes expression level before and after DBD plasma treatment

**Figure 6:**
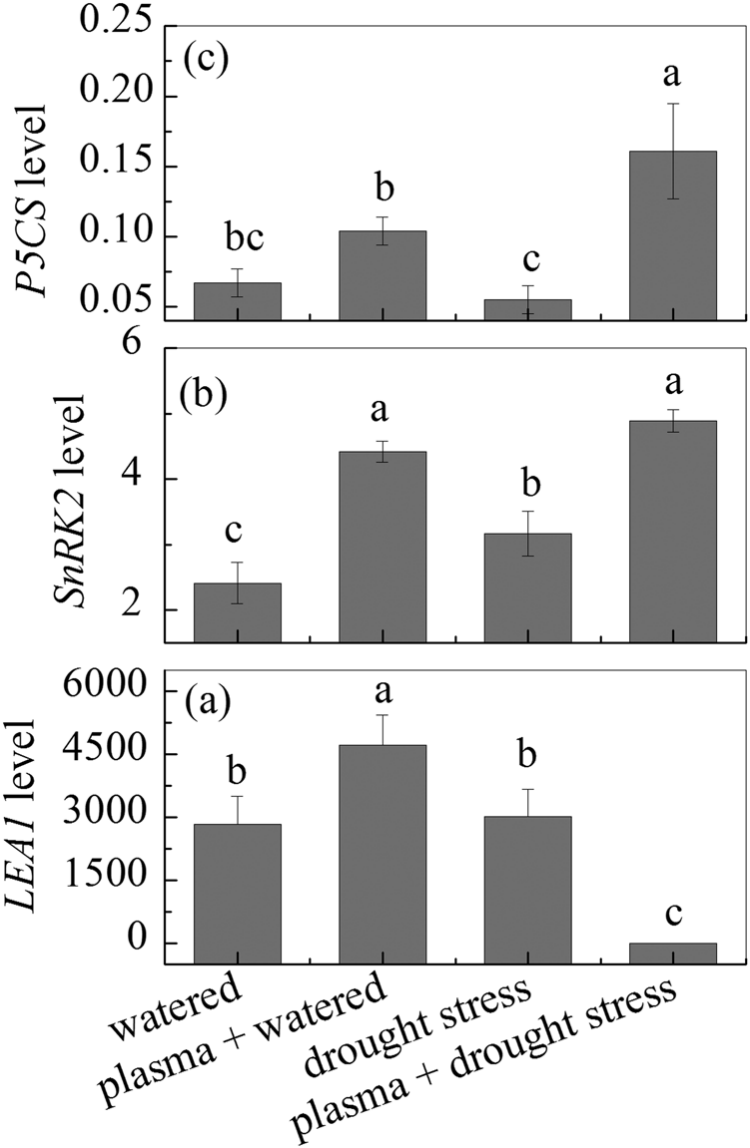
Expression patterns and relative expression level of *LEA1*, *SnRK2*, and *P5CS* in the wheat seedlings under drought stress before and after DBD plasma treatment. (The discharge voltage is 13.0 kV, and the DBD plasma treatment time is 4 min). Different lower case letters (**a**–**d**) indicate statistically significant differences at the *P* < 0.05 level).

The results of the drought resistant related genes (*LEA1*, *SnRK2*, *and P5CS*) expression levels under drought stress after the DBD plasma treatment are shown in Fig. 6. The DBD plasma treatment decreased the *LEA1* expression level under drought stress after the DBD plasma treatment, as shown in Fig. 6a; whereas the *SnRK2 and P5CS* expression levels under drought stress after the DBD plasma treatment were both enhanced, as shown in Fig. 6b and c.

### Effects of DBD plasma treatment on the morphology and structure of wheat seed coat

**Figure 7:**
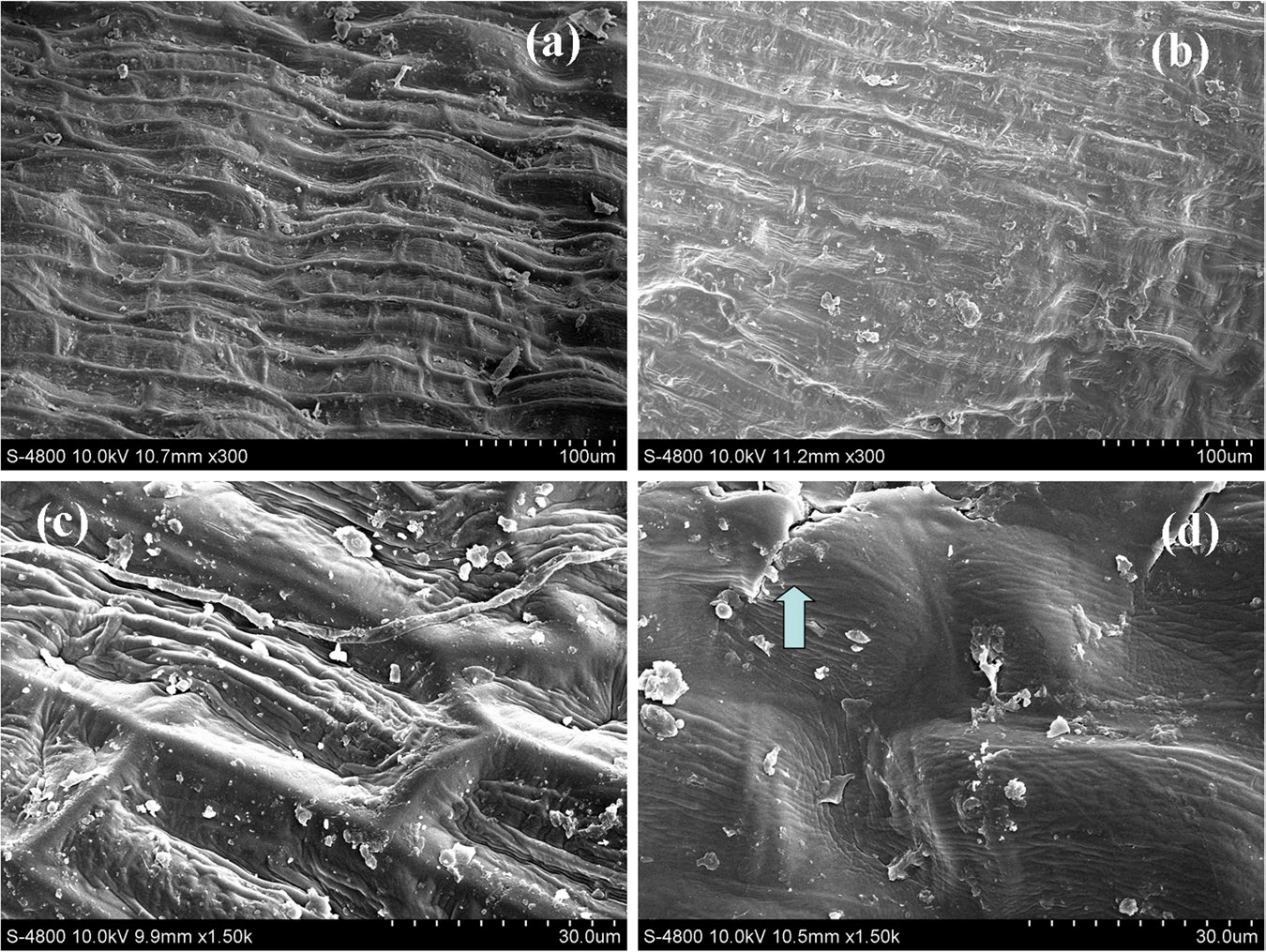
SEM photographs of wheat seed. (**a**) without DBD treatment, 300 magnification; (**b**) with DBD treatment, 300 magnification; (**c**) without DBD treatment, 1500 magnification; (**d**) with DBD treatment, 1500 magnification. The arrow in the figure indicates cracks in the seed coat. The discharge voltage is 13.0 kV, and the DBD treatment time is 4 min).

The surface morphology of the wheat seed coat before and after the DBD plasma treatment is shown in Fig. 7. As observed in Fig. 7a, many square mesh structures were clearly observed and the boundaries of these square mesh structures were also identified before the DBD plasma treatment; however, the boundaries of these square mesh structures became fuzzy and were quite difficult to identify after the DBD plasma treatment as shown in Fig. 7b. Furthermore, cracks were observed on the seed coat after the DBD plasma treatment as shown in Fig. 7d.

The elementary composition of the wheat seed coat before and after the DBD plasma treatment is shown in Fig. S2. No new element was observed after the DBD plasma treatment (Fig. S2b) compared to that before the DBD plasma treatment (Fig. S2a); however, the ratio of O/C was enhanced after the DBD plasma treatment.

**Figure 8:**
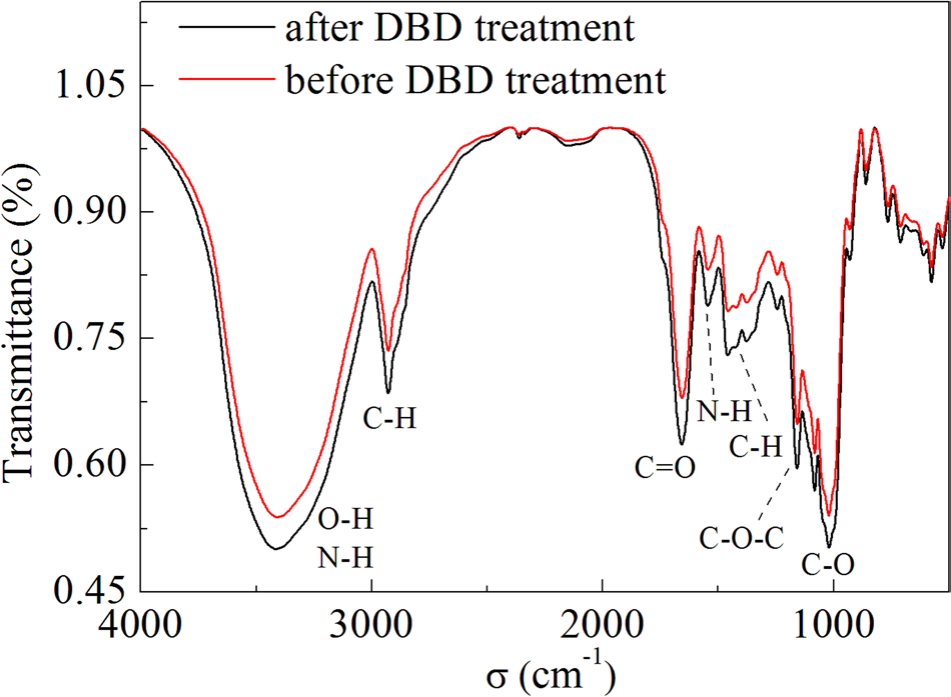
FTIR spectra of the wheat seed coat before and after DBD plasma treatment. (The discharge voltage is 13.0 kV, and the DBD treatment time is 4 min).

FTIR spectra of the wheat seed coat before and after the DBD plasma treatment are shown in Fig. 8. The intensities of some bands at 3413 cm^−1^, 2941 cm^−1^, 1664 cm^−1^, 1544 cm^−1^, 1155 cm^−1^, and 1016 cm^−1^ increased to various extents after the DBD plasma treatment, compared to those before the DBD plasma treatment.

## Discussion

### Relationship of DBD plasma treatment and physiological metabolic activities

Drought is one of the major constraints limiting crop growth. In the present study, we investigated the effects of the DBD plasma treatment on germination and seedling growth, osmotic adjustment ability, membrane lipid peroxidation, ROS, ABA, and antioxidant enzyme activities in the wheat seeds under drought stress.

First, exposure of wheat seeds to the DBD plasma showed some stimulating effects with respect to the germination potential and germination rate (Table 1). Previous research reported that DBD plasma treatment with an appropriate energy level enhanced *Andrographis paniculata* germination potential^15^. In our previous research, we also found that the DBD plasma treatment could enhance the wheat seed germination potential, germination rate, germination index and vigor index^20^. Li *et al*. reported that oilseed rape seed germination performance decreased under drought stress, whereas its germination rate and vigor index under drought stress were enhanced after a cold plasma treatment^16^. In our study, the DBD plasma treatment markedly increased the wheat seed germination potential and germination rate in the drought stress condition, and these results suggest that the DBD plasma treatment could alleviate the adverse effects of drought stress on wheat seed germination. In addition, the germination index of drought resistance was generally considered as an index to characterize drought resistance, and seeds with strong drought resistance usually had high values on the germination index of resisting drought^21^. In this study, we found that the DBD plasma treatment improved the germination index of drought resistance of the wheat seed (Table 1), which indicated that the drought resistance of the wheat seed after the DBD plasma treatment was enhanced.

The growth situation of plants could influence their resistance to adversity and improved growth was able to enhance their resistance. Liu *et al*. reported that *Triticum aestivum L*. seedling growth indices decreased under drought stress^22^. Previous studies has demonstrated that plasma treatment could promote oilseed rape and tomato seedling growth^11,16^, and it could also improve tomato and maize seedling growth under disease and cold stress, respectively^13,23^. Li *et al*. found that the root and shoot length of the oilseed rape seedlings decreased under drought stress, whereas they were enhanced after a cold plasma treatment^16^. DBD plasma treatment markedly increased the root and shoot length of the wheat seedlings under drought stress (Table 1), and these results indicated that the alleviation effects to drought stress in the wheat growth after the DBD plasma treatment might be attributed to their improved growth. In addition, the improvement of seed water uptake was quite important to promote seed growth under drought stress^24^. Our previous research has demonstrated that the DBD plasma treatment could promote water uptake of wheat seed^20^.

To resist drought stress, many plants changed their osmotic adjustment abilities via accumulating proline and soluble sugar levels, which participated in osmotic protection^22,25^. Previous research has demonstrated that electromagnetic treatment could alleviate the adverse effects of drought stress on maize growth, which was partly attributed to the improvement of the proline content in maize seedlings^9^. Chen *et al*. reported that soluble sugar content in brown rice increased after a cold plasma treatment^26^. Li *et al*. found that the soluble sugar content in oilseed rape increased under drought stress, which was further enhanced after a cold plasma treatment and thus alleviated the adverse effects of drought stress on oilseed rape growth^16^. Similarly, in response to drought stress, the contents of the proline and soluble sugar in the wheat seedlings both increased in our study, and their contents were further enhanced after the DBD plasma treatment (Fig. 1); the enhancement in the proline and soluble sugar levels after the DBD plasma treatment were related to the improvement in SOD and CAT activities due to their positive correlation (Table 2). It is known that SOD can convert O_2_^−^, and CAT can convert H_2_O_2_ to O_2_ and H_2_O molecules, and thus oxidative damages from ROS would decrease. In addition, the increase in the soluble sugar level could also be partly attributed to the ABA because of the positive correlation between them. It is also known that the ABA could regulate the osmotic adjustment of the plants.

In fresh seeds, ROS were generally maintained at low levels by cooperative reactions of enzymatic and non-enzymatic antioxidative systems; whereas in dry seeds, a large number of ROS, such as H_2_O_2_ and O_2_^−^, would be generated through auto-oxidation reactions of lipids; the overproduction of ROS could result in oxidative stress damage to the membrane lipids^25,27^. MDA is the product of membrane peroxidation and it is usually measured as an indicator of lipid peroxidation and membrane damage under drought stress^16^. Yin *et al*. reported that the cold plasma treatment reduced the MDA content in tomato seedlings^27^. Tong *et al*. also found that the MDA content in *Andrographis paniculata* seedlings significantly decreased after an air plasma treatment^15^. Li *et al*. found that the MDA content in oilseed rape seedlings increased under drought stress, compared to that in a well-watered condition^16^; however, the MDA content significantly decreased under drought stress after a cold plasma treatment. Wan *et al*. reported that drought stress resulted in a significant increase in the MDA, H_2_O_2_ and O_2_^−^ contents in cucumber seedlings, whereas their contents all decreased after a caffeic acid pretreatment, and thus, the resistance of the cucumber seedlings to drought stress was enhanced^25^. Similarly, the MDA, H_2_O_2_, and O_2_^−^ contents in the wheat seedlings increased in response to the drought stress in our study, whereas their contents decreased after the DBD plasma treatment (Figs 2 and 3); these results suggested that the DBD plasma treatment could promote the wheat seed germination and seedling growth by preventing oxidative damage of ROS derived from drought stress, because the ROS and MDA contents were negatively correlated with the wheat seed germination and seedling growth (Table 2).

To reduce the oxidative damage of ROS, plants can adjust the activities of the antioxidant systems to improve their resistance to drought stress; in this case, the levels of antioxidant enzymes, such as SOD, POD, and CAT, would increase, which were usually used to characterize the antioxidant capacity^25,27^. Among the antioxidant enzymes, SOD can convert O_2_^−^ to H_2_O_2_ and this was further converted to O_2_ and H_2_O molecules by antioxidant enzymes such as CAT and POD. Previous studies have demonstrated that the POD activity in plants was enhanced after the cold plasma treatment^13,27^. The activities of CAT and SOD in plants could also be improved after the cold plasma treatment^16,19^. In our study, the DBD plasma treatment significantly increased the SOD, POD, and CAT activities in the wheat seedlings under drought stress (Fig. 4). These results implicated that the DBD plasma treatment could enhance the ability of scavenging the ROS via improvement of the activities of the antioxidant enzymes in the wheat seedlings under drought stress, and thus reduce oxidative damages and help to maintain normal physiological metabolic activities. This maintenance phenomenon could also be confirmed via the increase in proline and soluble sugar levels due to the positive correlations among SOD, CAT, soluble sugar, and proline (Table 2), which increased the osmotic adjustment abilities of the wheat seedlings.

ABA is an important signal factor in response to dehydration and it can regulate the water status of plants via stomatal conductance and induce genes involved in dehydration resistance^28–31^. Desikan *et al*. reported that ABA synthesis was enhanced under drought stress^28^. Hu *et al*. pointed out that ABA could induce antioxidant defense systems and suppressed ROS damages under drought stress^29^. Previous research has also demonstrated that water stress-induced avoidance mechanisms were dependent on ABA synthesis, and the ABA could activate some enzymes, regulated the osmotic adjustment via adjusting proline transport to the root tip^30^, and improved the roots hydraulic conductivity via inducing gene expression for aquaporin synthesis^31^. In our study, the ABA levels were enhanced under drought stress, and it was further improved after the DBD plasma treatment (Fig. 5); there was a positive correlation between the ABA and soluble sugar and POD (Table 2). These results suggested that the DBD plasma treatment promoted ABA accumulation, and then helped to regulate the antioxidant enzyme defense system and enhanced its osmotic adjustment abilities, and thus reduced oxidative damages of ROS.

Two classes of drought induced genes have been reported in wheat, that is, functional gene such as *LEA*, and regulation genes such as *SnRK* and *P5CS* ^22^. Plant *LEA* gene had lots of important functions in plant growth and in protecting cell membrane and structure of plants from water deficit^32^; and it could improve tolerance to desiccation via suppressing protein aggregation and inactivation, stabilizing membranes, and maintaining enzymatic activity under drought stress^33^. Liu *et al*. found that the *LEA* expression level in *Triticum aestivum* L. under drought stress decreased after alginate oligosaccharides addition, and they deduced that exogenous alginate oligosaccharides could induce *LEA* gene to resist the damage from drought stress^22^. Goyal *et al*. reported that the *LEA* gene might act as a molecular chaperone, helping to prevent the formation of damaging protein aggregates under drought stress^33^. Tiwari *et al*. reported that RA-inoculation comparatively repressed the *LEA* gene expression under drought stress, suggesting its important roles in drought stress alleviation^34^. Jiang *et al*. found that there existed some relationship between *LEA* gene expression and activity of ROS scavenging enzymes, such as SOD, POD, and CAT^35^. Generally, the mechanisms for *LEA* functions mainly contained chaperone-like action and molecular shield activity, via binding to interaction partners accompanied by a folding transition, such as disorder-to-a mostly α -helix conformation upon drying^36,37^. In our study, the *LEA1* expression level in the wheat under drought stress also decreased after the DBD plasma treatment, this result suggested that the DBD plasma might regulate functional gene to resist drought stress for the wheat at early period. The enzymatic activity such as SOD, POD, and CAT were all improved under drought stress after the DBD plasma treatment (Fig. 4), this result suggested that the *LEA1* gene maintained the enzymatic activity. *SnRK* gene encoded a drought-induced putative protein kinase belonging to the *SnRK* subfamily; it could participate in complicated network responding to ABA signaling pathway^38^; and the *SnRK* gene could also regulate starch biosynthesis genes such as sucrose synthase and ADP-glucose pyrophosphorylase^39^. Seiler *et al*. reported that *SnRK* gene was up-regulated in the developing seeds under drought stress^40^. Liu *et al*. found that exogenous alginate oligosaccharides induced *SnRK* gene transcription to improve capacity of drought resistance under drought stress, and they attributed these changes to the involving of alginate oligosaccharides in ABA signaling pathway by stimulating ABA synthesis^22^. The *SnRK2* expression level in the wheat under drought stress increased after the DBD plasma treatment in our study, and the ABA content also increased after the DBD plasma treatment (Fig. 5), these results suggested that the DBD plasma treatment might involve in ABA signaling pathway to induce *SnRK2* gene transcription and to enhance the drought resistance of the wheat. *P5CS* gene was a typical gene for proline synthesis, and it usually increased under drought stress^41^; the *P5CS* activity represented a rate-limiting step in proline biosynthesis, which was controlled at the level of P5CS transcription and through feedback inhibition of P5CS by proline^42^. The *P5CS* expression level in the wheat under drought stress increased after the DBD plasma treatment in our study, and the proline content also increased after the DBD plasma treatment (Fig. 1a), these results suggested that the DBD plasma treatment might induce *P5CS* gene transcription to adjust the osmotic balance of the wheat. Liu *et al*. also reported that alginate oligosaccharides addition could enhance the drought resistance of *Triticum aestivum* L., and the *P5CS* expression levels increased after the alginate oligosaccharides addition^22^.

### Roles of DBD plasma treatment

When the discharge plasma occurs in air atmosphere, N_2_ and O_2_ molecules are excited and ionized by high-energy electrons, and then some active species, such as ·N radicals, N_2_^+^, and ·O radicals, would be generated, as shown in reactions 1~4^43^. N-containing species and O radicals were detected in the DBD plasma system using optical emission spectroscopy (Fig. S3).

1 $${e}+{{\rm{N}}}_{2}\to 2{e}+{{\rm{N}}}_{2}^{+}$$

2 $${e}+{{\rm{N}}}_{2}\to {e}+2\cdot {\rm{N}}$$

3 $${e}+{{\rm{O}}}_{2}\to 2\cdot {\rm{O}}+{e}$$

4 $$\cdot N+{{\rm{O}}}_{2}\to {\rm{NO}}+\cdot {\rm{O}}$$

It has been proven that reactive ions and radicals generated by cold plasma could etch and penetrated into the seed coat, which probably impacted some physiological actions in plants^44–47^. Sera *et al*. reported that active species generated in cold plasma could penetrate into the wheat and oat seeds, and then affected the contents of the phenolic compounds in it^12^. Filatova *et al*. reported that plasma etching effects exhibited significant roles in stimulating the biochemical processes of seeds and affecting seed germination^44^. The etching effects of the wheat seeds were also observed after the DBD plasma treatment in our study (Fig. 7), and these etching effects probably promote wheat seed germination, seedling growth, and some physiological and metabolic activities. In addition, previous research has demonstrated that ultraviolet radiation could alleviate drought-induced cell membrane damages to *Picea asperata* by reducing the level of lipid peroxidation^48^. Bright ultraviolet light was also observed in our experiment (Fig. S3), which might also participate in the stimulation of wheat seed germination, seedling growth, and some physiological and metabolic activities.

The actions of these active species on the seed could also enhance its wettability due to the oxidation of the seed surface by the active species, which then promoted the water uptake and thus benefited seed germination^20,49^. The oxidation of the wheat seed coat by the DBD plasma treatment could be confirmed via the increase in the ratio of O/C in Fig. S2. Some changes in the characteristic absorption peaks at 3413 cm^−1^, 2941 cm^−1^, 1664 cm^−1^, 1544 cm^−1^, 1155 cm^−1^, and 1016 cm^−1^ in the FTIR spectra of the wheat seed coat after the DBD plasma treatment were also observed (Fig. 8), and these absorption peaks were assigned to the bonded –OH group or N–H group in amino acids and nucleotides, the C–H stretching vibration of lipids, the C = O or C–N stretching vibration of proteins, the N–H stretching vibration of proteins, the C–O–C stretching vibration of lipase, and the C–O stretching vibration of polysaccharose, respectively^50,51^. These changes in the absorption peaks also demonstrated that the chemical structures of the wheat seed coat were changed by the DBD plasma treatment. In our previous research, we found that the DBD plasma treatment significantly promoted water absorption capacity and the relative electroconductivity of the wheat seeds^20^; therefore, the DBD plasma-treated wheat seed could resist the negative effects of the water deficit due to its strengthened water absorption capacity and permeability. Then, the osmotic adjustment substances (proline and soluble sugar), ABA content, and antioxidant enzyme activities were all enhanced, which could alleviate the damages of oxidative stress. Sera *et al*. reported that the wetting properties of oat and wheat seeds were promoted after a microwave plasma treatment, as well as their germination^12^. Similar phenomena were also observed by Bormashenko *et al*. in whose research the water absorption capacity and germination rate of oats were enhanced after a radiofrequency plasma treatment, and they attributed these results to the oxidation of the seed surface by the plasma^49^.

In summary, PEG-induced drought stress could cause significant oxidative damage to wheat seed germination and seedling growth, which were confirmed by the increased H_2_O_2_ and O_2_^−^ contents, more MDA generation, and enhanced proline and soluble sugar contents; whereas the DBD plasma treatment alleviated the oxidative damages to the wheat seed germination and seedling growth under drought stress. The DBD plasma treatment enhanced the oxidation resistance of the wheat seeds under drought stress via regulating functional genes *LEA1* and regulation genes *SnRK2* and *P5CS*, accumulating ABA, promoting the activities of antioxidant enzymes, and accumulating the proline and soluble sugar levels. Then, the MDA accumulation was reduced and the stability of the membrane structure was protected, and thus, wheat seed germination and seedling growth were enhanced. Due to the etching effect and surface modification by high-energy electrons, reactive ions, radicals, and ultraviolet irradiation of the DBD plasma, the permeability and water absorption capacity of the wheat seeds were promoted, which then benefited its germination and growth. However, further investigation on the effects of DBD plasma treatment on wheat growth and yield under drought stress should be conducted to provide an integrated strategy for enhancing its resistance to drought stress.

## Materials and Methods

### Wheat seed sample

Wheat seeds (Xiaoyan 22) were purchased from the Seed Research Institute of Northwest A&F University, China, and they were air dried, cleaned, and stored at 0–4 °C in a refrigerator; and the relative water content of the seeds was 10%.

### DBD plasma system and seed treatment

**Figure 9:**
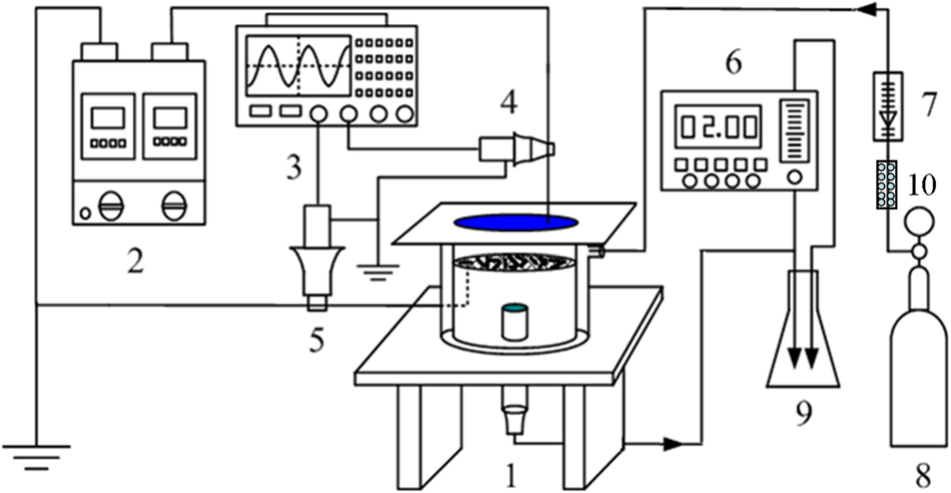
Schematic diagram of the discharge plasma system for seed treatment (1. reactor; 2. power source; 3. oscilloscope; 4. high voltage probe; 5. current probe; 6. ozone tester; 7. flowmeter; 8. gas source; 9. potassium iodide solutions; and 10. silicagel column).

The DBD plasma experimental setup for the seed treatment was illustrated in Fig. 9. The power supply was an alternating current with a discharge voltage range of 0–50 kV and a frequency of 50 Hz. The reactor vessel was made of a Plexiglas^TM^ cylinder (100 mm inner diameter and 8 mm height). A stainless-steel plate (120 mm diameter and 2 mm thickness) was used as the high voltage electrode, which was covered by a quartz glass piece (1.5 mm thickness and 180 mm diameter) as a dielectric barrier. The ground electrode was a metal net of 40 mesh. The distance between the quartz glass piece and the metal net was 8 mm. In each treatment, 50 wheat seeds were placed on the ground electrode and then treated by DBD plasma. Dry air (passing through a silicagel column to remove water molecules) was injected into the reactor vessel as the carrier gas. The voltage for the seed treatment was 13.0 kV (peak voltage), and the treatment time was 4 min. Each treatment was replicated three times. The typical voltage and current waveforms obtained in the DBD plasma system were shown in Fig. S4 in the supporting information (SI). Gaseous ozone generated in the DBD plasma system was trapped in potassium iodide solutions, and the detailed measurement method was reported by Suarasan *et al*.^43^; gaseous NO_2_ concentration was measured using Griess-Saltzman method as reported by Mendiara *et al*.^52^. The gaseous ozone was calculated as its mass divided by gas sampling volume under standard conditions, as well as NO_2_ concentration. In the present research, the gaseous ozone and NO_2_ concentrations were 1.17 mg L^−1^ and 0.11 mg L^−1^, respectively.

### Seed germination tests

Both controlled and DBD plasma-treated seeds were soaked in deionized water for 5 h, disinfected for 2 min by 70% alcohol, and then followed with a rinse in autoclaved distilled water and prepared for germination. The germination tests were divided into four groups; the single well-watered group (watered), single drought stress group (drought stress), plasma-treated and well-watered group (plasma + watered), and plasma-treated and drought stress group (plasma + drought stress). For the “plasma + watered” and “watered” groups, the germination tests were conducted in petri dishes (9 cm) containing two layers of filter paper and 10 mL of autoclaved distilled water; for the “plasma + drought stress” and “drought stress” groups, 10 mL of autoclaved distilled water was replaced with 10 mL of 15% (w/v) PEG-6000 solutions with an osmotic potential of −0.8 MPa as drought stress. The seeds from each treatment were tested on three petri dishes with 50 seeds per dish. During germination, autoclaved distilled water or 15% (w/v) PEG-6000 solutions was added every other day to maintain constant moisture. The seeds were incubated in a germination incubator at 20 °C, a 12 h light/12 h dark photoperiod a photo flux density of 120 µmol m^−2^ s^−1^, and 70% air humidity. The radicle protrusion at 1 mm was recorded as the criterion for germination. The seeds were incubated in the germination incubator for 4 d, and physiology and biochemistry indexes were calculated on the 4^th^ day of planting. The germination characteristics were calculated using the following equations^15^:

5 $${\rm{GP}}\,( \% )=\frac{{\rm{number}}\,{\rm{of}}\,{\rm{seeds}}\,{\rm{germinated}}\,{\rm{in}}\,{\rm{1}}\,{\rm{d}}}{{\rm{total}}\,{\rm{number}}\,{\rm{of}}\,{\rm{seeds}}}\times 100\, \% $$

6 $${{\rm{GR}}}_{{\rm{n}}}( \% )=\frac{{\rm{number}}\,{\rm{of}}\,{\rm{seeds}}\,{\rm{germinated}}\,{\rm{in}}\,{\rm{n}}\,{\rm{d}}}{{\rm{total}}\,{\rm{number}}\,{\rm{of}}\,{\rm{seeds}}}\times 100\, \% $$

where GP was the germination potential and it was calculated on the 1^st^ day of planting; GR_n_ was the germination rate on the n^th^ day.

To evaluate the drought resistance of the wheat seeds, the seeds were incubated in the germination incubator for 8 d, and the germination index of drought resisting (GIDR) was calculated using the following equations^53^:

7 $${\rm{GI}}=1\times {{\rm{GR}}}_{{\rm{2}}}+0.75\times {{\rm{GR}}}_{4}+0.50\times {{\rm{GR}}}_{6}+0.25{{\rm{GR}}}_{8}$$

8 $${\rm{GIDR}}={{\rm{GI}}}_{{\rm{DS}}}/{{\rm{GI}}}_{{\rm{CK}}}$$

where GI was the germination index; GR_2_, GR_4_, GR_6_, and GR_8_ were the germination rates on the 2^nd^, 4^th^, 6^th^, and 8^th^ day of planting, respectively; and GI_DS_ and GI_CK_ were the germination index under drought stress and the control group, respectively.

### Root and shoot length measurement

Fifteen seedlings from each petri dish were randomly collected to measure the total root length and total shoot length on the 4^th^ day of planting. The total root length and total shoot length of these seedlings were measured using a Vernier caliper; for the root length measurement, all of the roots of one seedling were measured. The root length in the later results represented the total root length of fifteen seedlings.

### Proline and soluble sugar measurement

Proline was measured using the method provided by Bates *et al*.^54^. The frozen coleoptiles were homogenized with 3% sulfosalicylic acid, and the mixtures were placed in a boiling water bath for 10 min, and centrifuged at 7000 rpm for 15 min at 4 °C, and then the supernatant (recorded as crude liquid) was used for proline measurement. The supernatant would be stored at 0–4 °C in a refrigerator if it could not be used immediately, and the maximum storage time was 36 h. For proline measurement, 2 mL of the above supernatant was mixed with 2 mL of acetic acid and 2 mL of acid ninhydrin, and the mixtures were placed in a water bath for 30 min at 98 °C. After cooling, the mixtures were extracted with 5 mL of toluene and then the absorbance of the extracting solution was measured at 520 nm. The proline concentration was expressed as the μmol g^−1^ fresh weight of the coleoptiles.

Soluble sugar was measured using the method provided by Ci *et al*.^55^. The frozen coleoptiles were homogenized with 80% alcohol, and the homogenate was washed with 80% alcohol three times; the homogenate was placed at room temperature for 30 min and centrifuged at 4 °C, and then the supernatant (recorded as crude liquid) was used for soluble sugar measurement. The supernatant would be stored at 0–4 °C in a refrigerator if it could not be used immediately, and the maximum storage time was 36 h. For soluble sugar measurement, 2 mL of the above supernatant was mixed with 3 mL of anthrone and the mixtures were placed in a boiling water bath for 10 min. After cooling, the absorbance of the mixtures was measured at 620 nm. The soluble sugar content was expressed as the mg g^−1^ fresh weight of the coleoptiles.

### MDA measurement

MDA was measured using the method provided by Liu *et al*.^22^. The frozen coleoptiles were homogenized with 0.1% of trichloroacetic acid and centrifuged at 10000 rpm for 15 min at 4 °C, and then the supernatant (recorded as crude liquid) was used for MDA measurement. The supernatant would be stored at 0–4 °C in a refrigerator if it could not be used immediately, and the maximum storage time was 36 h. For MDA measurement, 2 mL of the above supernatant was mixed with 2 mL of thiobarbituric acid, and the mixtures were placed in boiling water for 15 min. After cooling in ice-bath, the mixtures were centrifuged and the absorbance of the supernatant was measured at 532 nm, 600 nm, and 450 nm. The MDA content was expressed as the μmol g^−1^ fresh weight of the coleoptiles.

### H_2_O_2_ and O_2_^−^ measurement

The H_2_O_2_ concentration was measured using the method provided by Bai *et al*.^56^. The frozen coleoptiles were homogenized with ice-cold acetone, and centrifuged at 10000 rpm for 15 min at 4 °C, and then the supernatant (recorded as crude liquid) was used for H_2_O_2_ measurement. The supernatant would be stored at 0–4 °C in a refrigerator if it could not be used immediately, and the maximum storage time was 36 h. For H_2_O_2_ measurement, 2 mL of the above supernatant was mixed with 2 mL of 5% TiSO_4_ solutions. Then 0.5 mL of a 17 mol L^−1^ ammonia solution was added, and the mixtures were centrifuged after the reactions for 10 min. The precipitate was washed with ice-cold acetone three times to remove the chlorophyll and dissolved in a 1 mol L^−1^ H_2_SO_4_ solution, and then the absorbance of the solutions was measured at 410 nm. The H_2_O_2_ concentration was expressed as the μmol g^−1^ fresh weight of the coleoptiles.

The O_2_^−^ concentration was measured using the method provided by Bai *et al*.^56^. The frozen coleoptiles were homogenized with 4 mL of 65 mmol L^−1^ of phosphate buffer solution (PBS, pH = 7.8) and centrifuged at 4 °C, and then the supernatant (recorded as crude liquid) was used for O_2_^−^ measurement. The supernatant would be stored at 0–4 °C in a refrigerator if it could not be used immediately, and the maximum storage time was 36 h. For O_2_^−^ measurement, 1.0 mL of the above supernatant was mixed with 0.9 mL of 65 mmol L^−1^ PBS and 0.1 mL of 10 mmol L^−1^ hydroxylamine hydrochloride, and the mixtures were placed in a water bath for 20 min at 25 °C. Afterwards, 1.0 mL of 17 mmol L^−1^ sulfanilamide and 1.0 mL of 7 mmol L^−1^ α-anaphthylamine were added to the above mixtures. The mixtures were placed in a water bath for 20 min at 25 °C, and then the absorbance of the solutions was measured at 530 nm. The O_2_^−^ concentration was expressed as the μmol g^−1^ fresh weight of the coleoptiles.

### Enzymatic activity measurement

The frozen coleoptiles were homogenized with 5% (w/v) polyvinylpyrrolidone, 1.2 mL of 100 mmol L^−1^ potassium phosphate buffer (pH = 7.0) containing 1 mmol L^−1^ EDTA and 1% Triton X-100; the homogenates were centrifuged at 10000 rpm for 20 min at 4 °C, and then the supernatant (recorded as crude enzymes) was used for the enzymatic activity measurement. The supernatant would be stored at 0–4 °C in a refrigerator if the enzymatic activity measurement could not be conducted immediately, and the maximum storage time was 36 h.

The SOD activity was measured using the method provided by Liu *et al*.^57^. A total of 20 µL of the crude enzymes was mixed with 1.0 mL of the reactants containing 50 mmol L^−1^ potassium phosphate buffer (pH = 7.8), 6.5 mmol^−1^ methionine, 50 µmol L^−1^ NBT, 10 µmol L^−1^ EDTA, and 20 µmol L^−1^ riboflavin, and 1.0 mL portions of the reactants without the crude enzymes were used as the control. The mixtures were homogenized under dark conditions and then irradiated for 5 min by fluorescent lamps. Afterwards, the absorbance of the mixtures was measured at 560 nm.

The POD activity was measured using the method provided by Chance and Maehly^58^. A total of 20 µL of the crude enzymes was mixed with 1.0 mL of reactants containing 50 mmol L^−1^ potassium phosphate buffer (pH = 7.0), 200 mmol L^−1^ H_2_O_2_, and 25 mmol L^−1^ guaiacol. Afterwards, the increased absorbance value of the mixtures was measured at 470 nm every 1 min.

The CAT activity was measured using the method provided by Chance and Maehly^58^. A total of 20 µL of the crude enzymes was mixed with 1.0 mL of reactants containing 50 mmol L^−1^ potassium phosphate buffer (pH = 7.0), and 10 mmol L^−1^ H_2_O_2_. Afterwards, the decreased absorbance value of the mixtures was measured at 240 nm every 1 min.

### ABA measurement

ABA extraction and measurement were conducted as described by Kelen and Ozkan^59^. The coleoptile samples were homogenized with 70% methanol and stirred overnight at 4 °C, and the extract was filtered through a Whatman filter and the methanol was evaporated in vacuum. The aqueous phase was then adjusted to pH 8.5 with 0.1 mmol L^−1^ phosphate buffer and shaken several times with ethyl acetate. Subsequently, the aqueous phase was adjusted to pH 2.5 with 1 mol L^−1^ HCl after the ethyl acetate was removed; it was shaken several times with diethyl ether, and then the aqueous phase was passed through waterless sodium sulfate. Finally, the diethyl ether phase was evaporated in a vacuum and the dry residue containing hormones were dissolved in 2 mL methanol and stored in vials at 4 °C prior to measurement. An HPLC system equipped with a C18 column (25 μm, 4.6 × 250 mm) was used to measure the ABA; the mobile phase was methanol-phosphoric acid buffer (35:65, v/v) with a total flow rate of 0.8 mL min^−1^, and the detection wavelength was set at 265 nm.

### Total RNA isolation and quantitative PCR (qPCR)

Total RNAs from coleoptiles of “plasma + watered”, “watered”, “plasma + drought stress”, and “drought stress” groups were isolated using an RNA extraction kit (Transgen, China). The cDNA was synthesized using MultiScribe reverse transcriptase (Transgen, China). Quantitative real-time RT-PCR (qRT-PCR) was performed using SYBR Premix Ex Taq II (Takara, China) on an iQ5 Real-Time PCR Detection System (BIO-RAD, USA). The expression of *LEA1* gene, *psbA* gene, Sucrose nonfermenting 1-related protein kinase 2 gene (*SnRK2*) and Pyrroline-5-Carboxylate Synthetase gene (*P5CS*) were determined using qRT-PCR. The *actin* gene was used as an internal reference. Three biological replicates were performed for these experiments. The specific primers of drought responsive genes were listed in Table S1.

### Composition analysis of the seed coat

FTIR spectroscopy (Nicolet NEXUS 470) was applied to characterize the functional groups of the seed coat. Sample discs were prepared by mixing 1 mg of the samples with 500 mg of KBr in an agate mortar and scanned at a range from 4000 to 400 cm^−1^; 100 scans were taken at a resolution of 4 cm^−1^. Scanning electron microscopy (SEM, S-4800, Hitachi) was used to characterize the morphology of the seed coat, and the backside of the seeds was selected for observation.

### Statistical analysis

All of the treatments were conducted with at least three replicates. The data in this study was recorded as the mean value ± standard deviation. The SPSS statistical software (Version 16.0) and one-way analysis of variance (ANOVA) were used to confirm the variability of the data and the validity of the results. Differences among the treatments were compared using Duncan’s multiple range tests at the 0.05 probability level. Correlations among the measured parameters were determined using the Pearson’s correlation coefficient by SPSS statistical software (Version 16.0).

## Electronic supplementary material

Supporting Information
