# Supplementary material for: Alleviation of adverse effects of drought stress on wheat seed germination using atmospheric dielectric barrier discharge plasma treatment
Source: Sci Rep. 2017 Nov 30;7:16680. doi: 10.1038/s41598-017-16944-8 (PMC5709406; doi:10.1038/s41598-017-16944-8)
Supplement: Supplementary file 1 — Supporting Information [file 41598_2017_16944_MOESM1_ESM.doc]

**Supporting Information**

**Alleviation of adverse effects of drought stress on wheat seed germination using atmospheric dielectric barrier discharge plasma treatment**

Qiao Guo^2,3^, Ying Wang^2,3^, Haoran Zhang^2,3^, Guangzhou Qu^2,3^, Tiecheng Wang^1,2,3*^, Qiuhong Sun^3^, Dongli Liang^2,3^

^1^State Key Laboratory of Soil Erosion and Dryland Farming on the Loess Plateau, Institute of Soil and Water Conservation, Northwest A&F University, Shaanxi Province 712100, PR China

^2^College of Natural Resources and Environment, Northwest A&F University, Yangling, Shaanxi Province 712100, PR China

^3^Key Laboratory of Plant Nutrition and the Agri-environment in Northwest China, Ministry of Agriculture, Yangling, Shaanxi 712100, PR China

*Corresponding author: Tiecheng Wang

E-mail: wangtiecheng2008@126.com

**4 Pages**

**4 Figures**

**1 Tables**

**
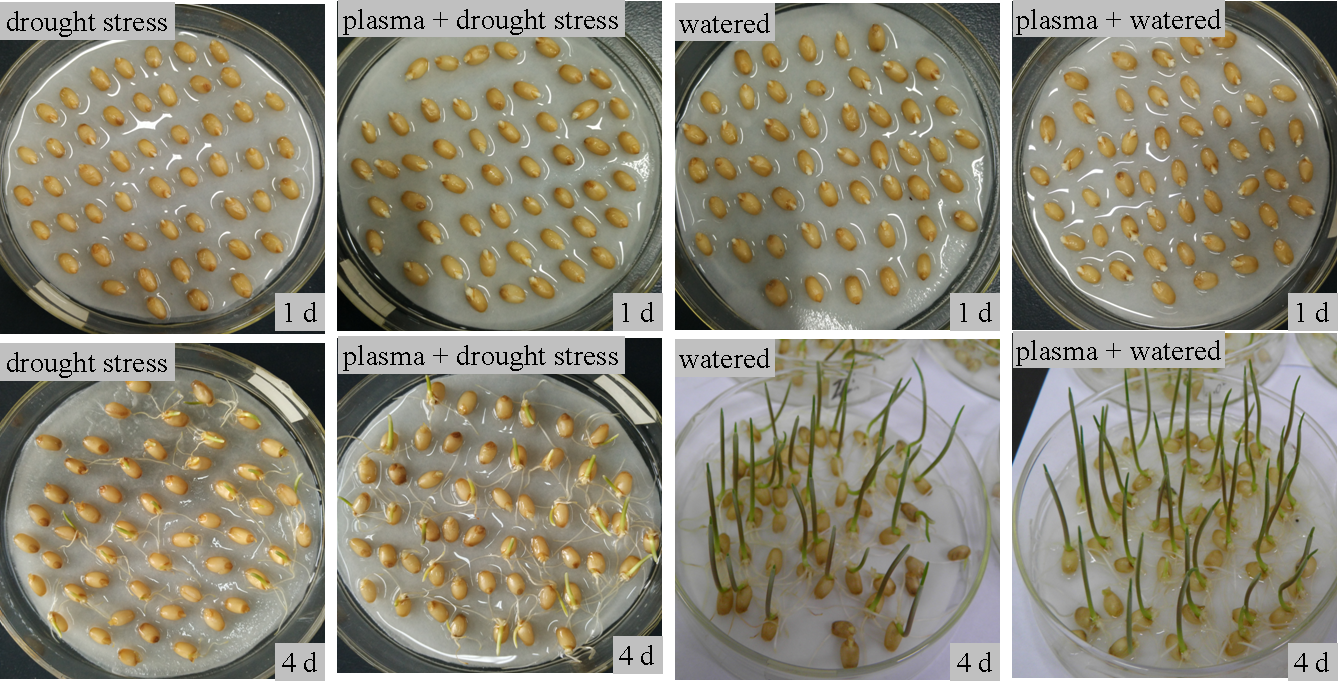
**

**Fig. S1** Wheat seed germination photos under drought stress and plasma treatment (1d: samples are collected on the 1^st^ day of planting; 4d: samples are collected on the 4^th^ day of planting. The discharge voltage is 13.0 kV, and the DBD treatment time is 4 min)


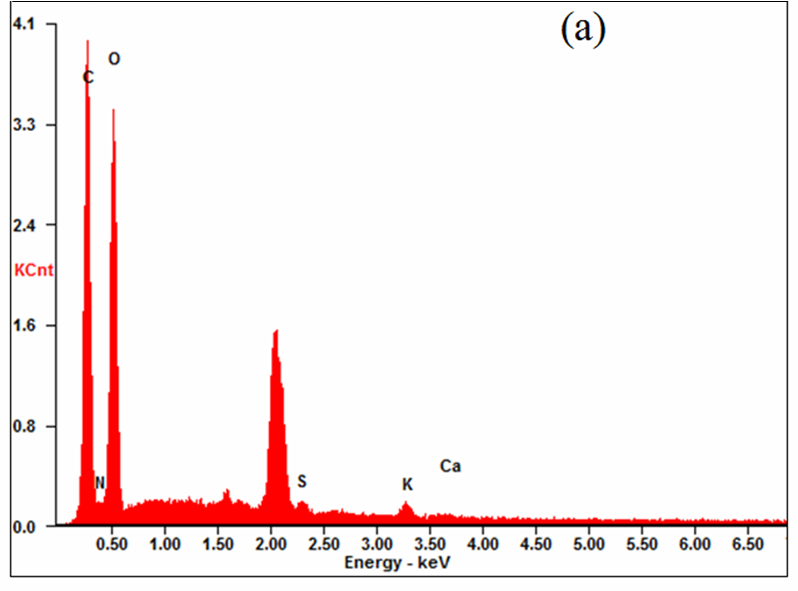


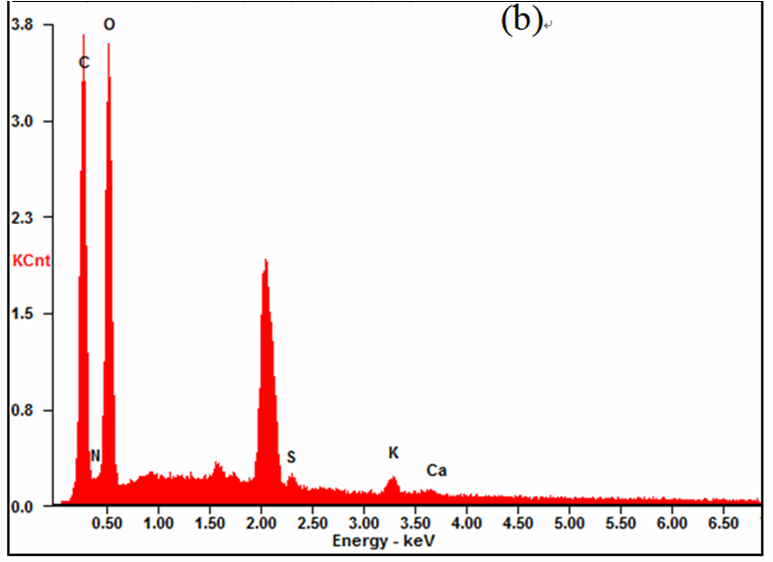


**Fig. S2** EDX photographs of wheat seed coat (a: without DBD treatment; b: with DBD treatment. The discharge voltage is 13.0 kV, and the DBD treatment time is 4 min)


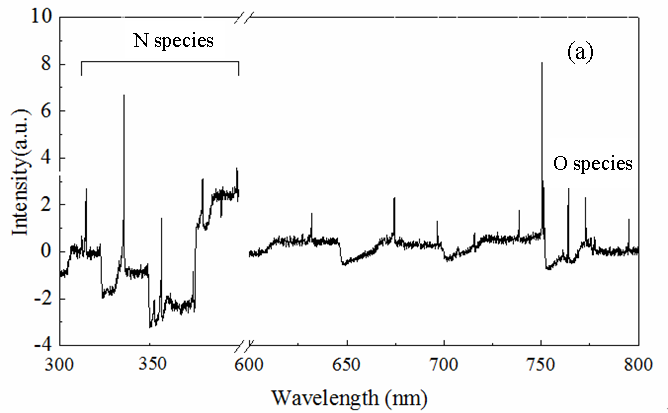


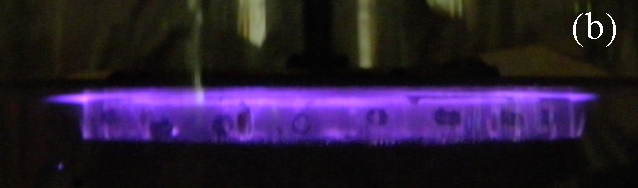


**Fig. S3** Typical emission spectrum and discharge photograph of the DBD system (a. emission spectrum; b. discharge photograph)

**Fig. S4** Typical voltage and current waveforms obtained in the DBD plasma system

**Table S1.** Primer sequences

| Primer | Sequences | Accession number or source | Annotation |
| --- | --- | --- | --- |
| *TaActin* | 5’-CTATCCTTCGTTTGGACCTT-3’  5’-AGCGAGCTTCTCCTTTATGT-3’ | NCBI (AB181991.1) | Internal reference gene |
| *TaLEA1* | 5’-AAGGACCAGACCGCCAGCAC-3’  5’-AAGGACCAGACCGCCAGCAC-3’ | NCBI (AY148490.1) | Late embryogenesis abundant proteins gene |
| *TaSnRK2* | 5’-GATTCAAGGTGGTCGTGC-3’  5’-TTCCCTCATCCTCGCTAA-3’ | NCBI (DQ343300.1) | SNF1-related protein kinase 2 gene |
| *TapsbA* | 5’-GTTCCCACTCACGACCCA-3’  5’-GACCGCAACTTCTGTATTTATT-3’ | NCBI (NC_002762.1) | Chlorophyll synthesis relevant gene |
| *TaP5CS* | 5’-TACAGCGGTCCACCAAGT-3’  5’-GCCACCTCTACCAACACG-3’ | NCBI (AY888045.1) | Δ^1^-pyrroline-5-carboxylate synthetase gene |
